# Modification of porcine plasma protein structure and flavor by ultrasound-assisted enzymatic hydrolysis

**DOI:** 10.1016/j.ultsonch.2026.108111

**Published:** 2026-10-12

**Authors:** Lingrui Li, Jingwei Pan, Tiantian Wu, Chuan Pang, Xin Guo, Shiling Lu, Jingyun Wang

**Affiliations:** a Key Laboratory of Characteristics Agricultural Product Processing and Quality Control (Co-construction by Ministry and Province), Ministry of Agriculture and Rural Affairs, School of Food Science and Technology, Shihezi University, Shihezi, Xinjiang 832000, China; b Key Laboratory for Food Nutrition and Safety Control of Xinjiang Production and Construction Corps, School of Food Science and Technology, Shihezi University, Shihezi, Xinjiang 832000, China; c Center for Livestock Product Processing and Quality Safety Control, School of Food Science and Technology, Shihezi University, Shihezi, Xinjiang 832000, China; d Xinjiang Qinghu Tiankang Food Co., Xinjiang Autonomous Region, Wujiaqu, China

**Keywords:** Porcine plasma protein, Ultrasound pretreatment, Protein structure, Flavor

## Abstract

This study evaluated the effects of ultrasound-assisted enzymatic hydrolysis on the structural and flavor properties of porcine plasma protein (PPP). Five proteases, pepsin, stem bromelain, ficin, neutral protease and papain, were used to hydrolyze porcine plasma protein, and their effects on the degree of hydrolysis (DH) were compared. The neutral protease-treated group exhibited the highest DH, reaching 11.74 ± 0.04%. Ultrasonic pretreatment (500 W, 5 min) further increased the DH to 17.43 ± 0.40% and improved sweetness and umami taste, as revealed by sensory evaluation and electronic tongue/nose analyses. Free amino acid analysis showed that total free amino acids in the ultrasound-assisted group reached 3515.81 ± 12.17 μg/g, with significant increases in umami- and sweetness-associated amino acids. Structural characterization using endogenous fluorescence, surface hydrophobicity, particle size/zeta potential, and scanning electron microscopy indicated that the combined effect of ultrasound and enzymatic hydrolysis induced protein unfolding, exposed hydrophobic regions, and enhanced enzyme accessibility. These changes included increased α-helix and random coil contents, reduced β-turn content, enhanced surface hydrophobicity, reduced particle size, and a more fragmented and porous microstructure. This study provides a theoretical basis for using ultrasound-assisted enzymatic hydrolysis to improve the umami taste of porcine plasma protein.

## Graphical abstract

**Figure:**
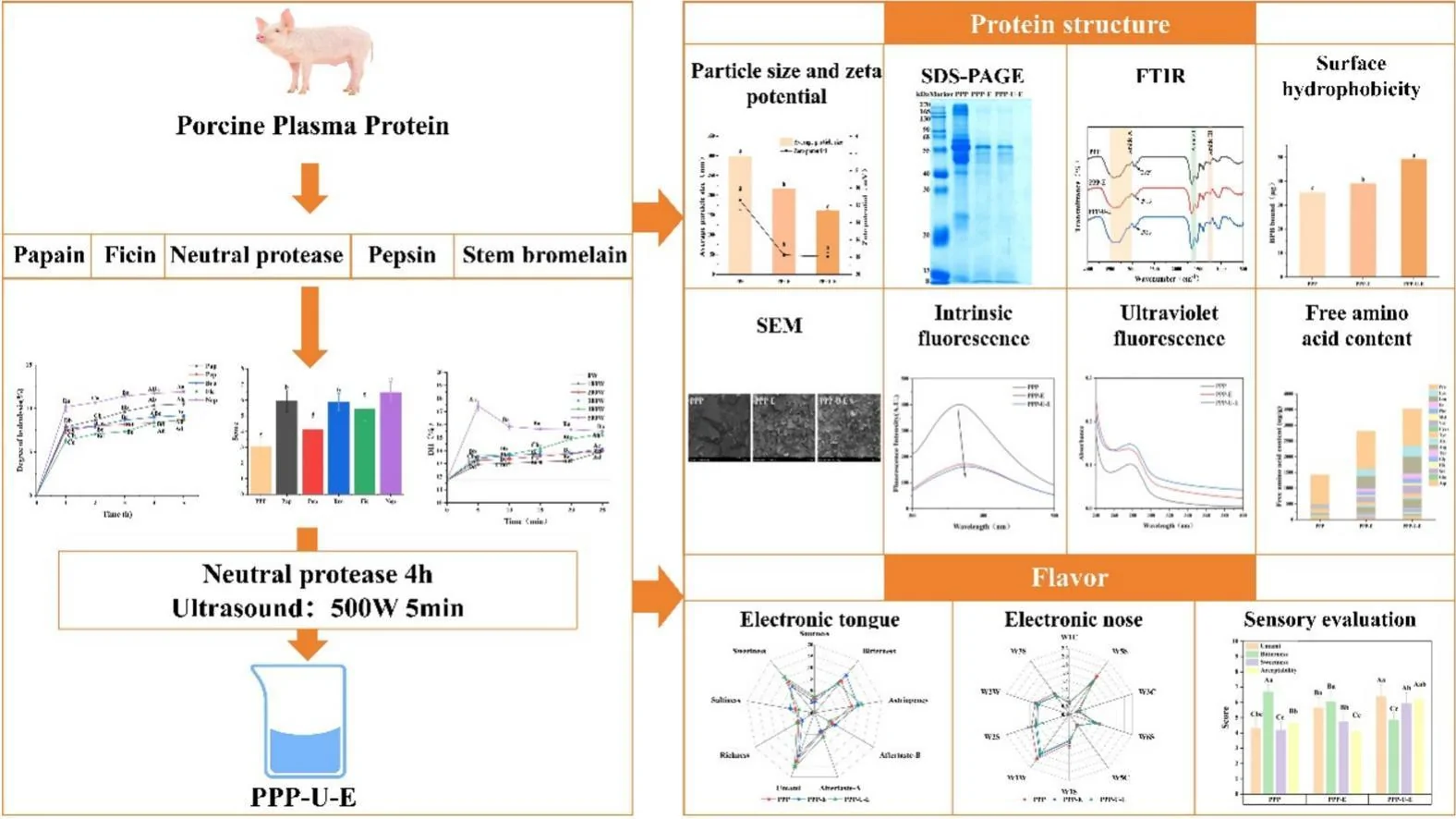

## 1 Introduction

Pig blood, a major byproduct of slaughterhouses, remains underutilized despite its abundance, often discarded or inefficiently processed [1]. It serves as a high-quality protein source, composed primarily of plasma proteins and hemoglobin. Plasma accounts for approximately 60% of whole blood volume, and porcine plasma protein (PPP) constitutes about 6–8% of plasma, mainly comprising serum albumin (50%–60%), globulins (40%–50%), and fibrinogen (1%–3%) [2], [3]. PPP exhibits favorable functional properties, including gelling, emulsifying, and foaming capabilities. Nevertheless, its use in foods is hindered by inherent unpleasant tastes, such as bloodiness and a metallic flavor [4], [5]. Therefore, enhancing palatability and enabling high-value utilization of PPP through protein modification techniques is essential.

Enzymatic hydrolysis is a well-established technique for enhancing protein flavor. This process involves the use of proteases to release peptides, which function as flavor precursors or mask undesirable flavors [6]. However, different proteases exhibit distinct cleavage sites and specificities, resulting in varying effects on protein flavor. Tang et al. reported that pepsin, trypsin, and neutral protease exhibit distinct enzyme specificity in generating different flavors during egg yolk hydrolysis [7]. However, targeted enzymatic hydrolysis studies for PPP flavor remain unexplored.

Conventional enzymatic hydrolysis methods are often limited by site shielding due to the spatial structure of proteins, which restricts cleavage efficiency and thereby limits the potential for optimizing the flavor of products [8]. Ultrasonication has recently emerged as an efficient coupling technology due to its cavitation and mechanical effects [9]. The cavitation effects and mechanical vibrations induced by ultrasonic treatment can disrupt the higher-order structures of proteins, thereby exposing enzymatic sites and enhancing enzyme-substrate interactions. These structural modifications accelerate the enzymatic reactions and improve hydrolysis efficiency [10]. Previous studies have demonstrated that parameters such as ultrasonic power (100–500 W) and duration (5–25 min) significantly influence the degree of protein hydrolysis and affect the flavor characteristics of the final product [11], [12]. Despite these findings, investigations into the enhancement of the flavor profile of PPP through ultrasonic-assisted enzymatic hydrolysis remain relatively limited.

Five enzymes were selected based on their documented hydrolytic activity toward proteins, as reported in previous studies: pepsin (Pep), stem bromelain (Bro), ficin (Fic), neutral protease (Nep), and papain (Pap) [13], [14], [15]. However, systematic studies on the umami-modifying effects of these specific enzymes on PPP remain limited. The effects of these five proteases on the degree of hydrolysis and umami of PPP were evaluated over different enzymatic digestion times. After selecting the optimal protease, ultrasonication was applied to investigate the combined effect of ultrasound and enzymatic hydrolysis on PPP flavor enhancement. This study provides a theoretical basis for targeted PPP flavor modification and offers new insights into the high-value utilization and flavor regulation of livestock byproduct proteins.

## 2 Materials and methods

### 2.1 Materials

The PPP used in this study was sourced from Shaanxi Jiyu Jintai Biotechnology Co., Ltd. (Yangling, Shaanxi, China). Fic (100,000 U/g) was procured from Guangdong Huasheng Food Technology Co., Ltd. (Shantou, Guangdong, China). Pap (100,000 U/g) and Bro (500,000 U/g) were obtained from Guangzhou Heykang Food Technology Co., Ltd. (Guangzhou, Guangdong, China). Pep (3,000,000 U/g) was sourced from Shanghai Yien Chemical Technology Co., Ltd. (Shanghai, China). Nep (50,000 U/g) was procured from Chengdu Maohesheng Technology Co. Ltd. (Chengdu, Sichuan, China). All chemicals and solvents used for analysis were of an appropriate grade.

### 2.2 Enzymolysis of PPP

PPP powder was dissolved in 0.01 M PBS (pH 7.0) to give a 40 mg/mL protein solution. The temperature and pH of the solution were subsequently adjusted in a water bath to the optimal conditions specified for each protease. The protease was added under continuous stirring to achieve a final enzyme concentration of 4% (w/v) after equilibrium was reached. Five proteases were evaluated at their respective supplier-recommended conditions and an enzyme loading of 6,000 U/g substrate: Pep (37 °C, pH 3.0), Fic (50 °C, pH 5.7), Bro (55 °C, pH 7.0), Nep (40 °C, pH 7.5), and Pap (50 °C, pH 7.5). Hydrolysis was conducted for 1–5 h, with 0.1 M NaOH added dropwise to maintain the target pH. After hydrolysis, enzymes were denatured by heating at 100 °C for 15 min. Centrifugation of the hydrolysate (9,000 × *g*, 10 min) was followed by freeze-drying of the supernatant to obtain hydrolyzed PPP.

The degree of hydrolysis (DH) of PPP hydrolysate was measured by the OPA assay. Optimal conditions (Nep, 6,000 U/g, 4 h) were then selected based on DH and sensory evaluation to prepare PPP-E.

### 2.3 Ultrasound-assisted enzymolysis of PPP

Before enzymatic hydrolysis, PPP solution was pretreated with ultrasound using a cell disruptor operating at 25 kHz. Pretreatment was performed at varying power outputs (0, 100, 200, 300, 400, and 500 W) and durations (5, 10, 15, 20, and 25 min). To prevent overheating, ultrasound was applied in pulse mode with a 3 s on/3 s off cycle. 0 W represents the control subjected to enzymatic hydrolysis only, without ultrasonic pretreatment (PPP-E).

Following pretreatment, hydrolysis was performed for 4 h using the optimal protease identified (Nep, 40 °C, pH 7.5, enzyme loading of 6,000 U/g substrate). The resulting hydrolysate with the highest DH was selected and designated as PPP-U-E for subsequent analyses.

### 2.4 Sensory evaluation

Trained and screened assessors conducted the sensory evaluation. The panel consisted of 20 assessors (10 males and 10 females), aged 22–55 years. During screening, candidates were required to correctly identify basic taste solutions: 3.50 g/L monosodium glutamate (umami), 50 g/L sucrose (sweetness), and 0.15 g/L caffeine (bitterness). Formal evaluations were performed at 23 ± 2 °C using quantitative descriptive analysis. A 10-point scale was utilized to evaluate sensory attributes (umami, sweetness, bitterness, and overall acceptability). For umami, sweetness, and bitterness, intensity was rated as follows: 0–2 (extremely weak), 2–4 (weak), 4–6 (moderate), 6–8 (strong), and 8–10 (extremely strong). Overall acceptability was evaluated on the same 10-point scale, with descriptors ranging from extremely unacceptable to extremely acceptable.

Samples (1 mL each) were presented in randomly coded containers in a randomized order. Panelists retained each sample in the mouth for 10 s, expectorated, and rinsed with warm water. We enforced a minimum 2-min interval between samples to minimize sensory fatigue and carryover effects.

The Research Ethics Committee of Shihezi University approved all procedures. All participants provided informed consent before participation. Overall acceptability, evaluated after the intensity ratings of individual taste attributes, served as a comprehensive indicator of flavor harmony and palatability. Along with umami, sweetness, and bitterness scores, it provided a systematic characterization of the overall flavor profile.

### 2.5 Electronic tongue analysis

The taste profile was analyzed using an electronic tongue system (SA402B, Intelligent Sensor Technology Co., Ltd., Atsugi, Japan). The supernatant of each sample was used directly for measurement. A reference solution of 30 mM KCl and 0.3 mM tartaric acid was used.

### 2.6 Electronic nose analysis

An electronic nose (PEN3, Airsense Analytics GmbH, Schwerin, Germany) analyzed sample flavor profiles. For each measurement, 15 mL of sample was placed in a beaker, sealed with plastic wrap, and equilibrated at 25 °C for 15 min to stabilize the headspace. The system was equipped with ten metal oxide sensors targeting: W1C (aromatic compounds and benzenoids), W5S (nitrogen oxides), W3C (ammonia and aromatic compounds), W6S (hydrides), W5C (alkanes and aromatic compounds), W1S (methane), W1W (inorganic sulfides), W2S (alcohols, ethers, aldehydes, and ketones), W2W (aromatic and organic sulfur compounds), and W3S (long-chain alkanes).

### 2.7 Particle size and zeta potential

A Malvern Zetasizer (Malvern Panalytical Ltd., Malvern, UK) measured the particle size and zeta potential of samples diluted to 0.1 mg/mL. Measurements were performed at 25 °C with an equilibration time of 120 s.

### 2.8 Sodium dodecyl sulfate–polyacrylamide gel electrophoresis (SDS-PAGE)

SDS-PAGE used a separating gel of 12% and a stacking gel of 4%. Samples prepared at 5 mg/mL were denatured with loading buffer (100 °C, 5 min). Electrophoresis was carried out at 80 V until the samples reached the separating gel, followed by 120 V to finish.

### 2.9 Fourier transform infrared (FTIR) spectroscopy

The freeze-dried sample was mixed with KBr (1:400, w/w), thoroughly ground in a mortar, and then compressed into a thin pellet, after which FTIR spectra were recorded using a Bruker Vertex 70 V spectrometer (Bruker Ltd., Billerica, MA, USA). Data analysis was performed using OMNIC 32 and PeakFit 4.12 software.

### 2.10 Endogenous fluorescence spectroscopy

A fluorescence spectrophotometer (RF-2700, Hitachi High-Tech Corporation, Tokyo, Japan) was used to measure intrinsic fluorescence spectra of PPP, PPP-E, and PPP-U-E. Samples were prepared at 0.1 mg/mL in 0.1 M phosphate buffer (pH 7.0). Using an excitation wavelength of 280 nm, emission spectra were recorded from 300 to 500 nm with a slit width of 5 nm for both excitation and emission.

### 2.11 Surface hydrophobicity

Surface hydrophobicity was determined using the bromophenol blue (BPB) binding method as described by Chelh et al. [16] with slight modifications. Briefly, the sample (0.1 mg/mL in 0.1 M phosphate buffer, pH 7.0) was mixed with BPB solution (1 mg/mL) at a 5:1 ratio (v/v) and vortexed thoroughly. After centrifugation (4000 rpm, 10 min, 4 °C), the supernatant was diluted 10-fold with phosphate buffer. The absorbance was measured at 595 nm against a phosphate buffer blank. The amount of BPB bound to the protein was calculated using the following equation:

$$BPB\;bound\left(\mu g\right)=200\;\mu g\times \frac{A_{control}-A_{sample}}{A_{control}}$$

where A_control_ and A_sample_ are the absorbances of the control and sample, respectively, at 595 nm.

### 2.12 Ultraviolet–visible (UV–vis) absorption spectra

Samples were diluted to 0.1 mg/mL with 0.1 M phosphate buffer (pH 7.0), and UV–Vis spectra were recorded from 240 to 400 nm using a UV spectrophotometer (UV-2600, Shimadzu, Kyoto, Japan).

### 2.13 Scanning electron microscopy (SEM)

Microstructure was observed using an SU8010 SEM (Hitachi High-Tech Corporation, Tokyo, Japan) at an accelerating voltage of 2.0 kV. Samples were gold-coated on conductive tape and imaged at 100×.

### 2.14 Free amino acids (FAAs)

#### 2.14.1 Sample preparation

Each sample (1.0 g, precisely weighed) was mixed with 20 mL of water, soaked for 2 h, and subjected to ultrasonic extraction at 60 °C and 250 W for 1 h. The mixture was filtered under vacuum, and the filtrate was freeze-dried to a powder. The resulting powder was redissolved in water and diluted to a final volume of 10 mL. A blank control was prepared using the same procedure without sample addition.

#### 2.14.2 Chromatographic conditions

FAA content was determined by OPA and FMOC pre-column derivatization reversed-phase high-performance liquid chromatography with ultraviolet detection, using an Agilent 1200 HPLC system (Agilent Technologies Co., Ltd., California, USA).

Chromatographic separation was performed on a Welchrom HPLC C18 column (4.6 × 250 mm, 5 μm) at 40 °C. Mobile phase A was sodium acetate buffer (8 g/L sodium acetate trihydrate, containing 0.022% triethylamine and 0.5% tetrahydrofuran, pH 7.20). Mobile phase B was sodium acetate buffer (6 g/L sodium acetate trihydrate)–acetonitrile–methanol (20:40:40, v/v/v, pH 7.20). Both mobile phases were filtered through 0.45 μm membranes and degassed by ultrasonication before use. OPA derivatives were detected at 338 nm, and FMOC derivatives at 262 nm. The elution gradient was carried out for 35.5 min as follows: 0–27.5 min, 92% to 40% A at 1.0 mL/min; 27.5–31.5 min, 40% to 0% A with the flow rate increasing linearly from 1.0 to 1.5 mL/min; 31.5–32.0 min, 0% A at 1.5 mL/min; 32.0–34.0 min, 0% A with the flow rate decreasing linearly from 1.5 to 1.0 mL/min; 34.0–35.5 min, 0% to 92% A at 1.0 mL/min.

### 2.15 Statistical analysis

Each experiment was performed in triplicate (n = 3), and the results are presented as the mean ± standard deviation (SD). Data were analyzed using one-way analysis of variance (ANOVA) with IBM SPSS Statistics (version 27.0; IBM Corp., Armonk, NY, USA). Significant differences between groups were determined at a significance level of *P* < 0.05. OriginLab software (OriginLab Corporation, Northampton, MA, USA) was used for principal component analysis (PCA) and graph generation.

## 3 Results and discussion

### 3.1 Effects of different proteases on DH and umami characteristics of PPP

The five selected proteases (Nep, Pap, Bro, Fic, and Pep) were investigated to determine the effect of hydrolysis time on DH and umami characteristics of PPP. Fig. 1A depicts that DH increased with prolonged hydrolysis time for all proteases tested, indicating that each enzyme can effectively hydrolyze PPP. Notably, the DH of Nep, Pap, Bro, Fic, and Pep increased rapidly within the first 4 h, reaching 11.74 ± 0.04%, 10.33 ± 0.44%, 9.00 ± 0.12%, 8.34 ± 0.44%, and 8.32 ± 0.21%, respectively, before stabilizing. These findings suggest that enzyme activity is most efficient during the initial hydrolysis stage. This trend is consistent with the findings of Li et al. [14], who reported that protein hydrolysis proceeds rapidly within the first few hours before gradually entering a stationary phase. The neutral protease-treated group exhibited a significantly higher DH than the other protease-treated groups (*P* < 0.05), suggesting that neutral protease is the most effective enzyme for PPP hydrolysis. This enhanced performance may be attributed to the endopeptidase activity of Nep, which enables extensive cleavage of internal peptide bonds within proteins [13].

**Fig. 1:**
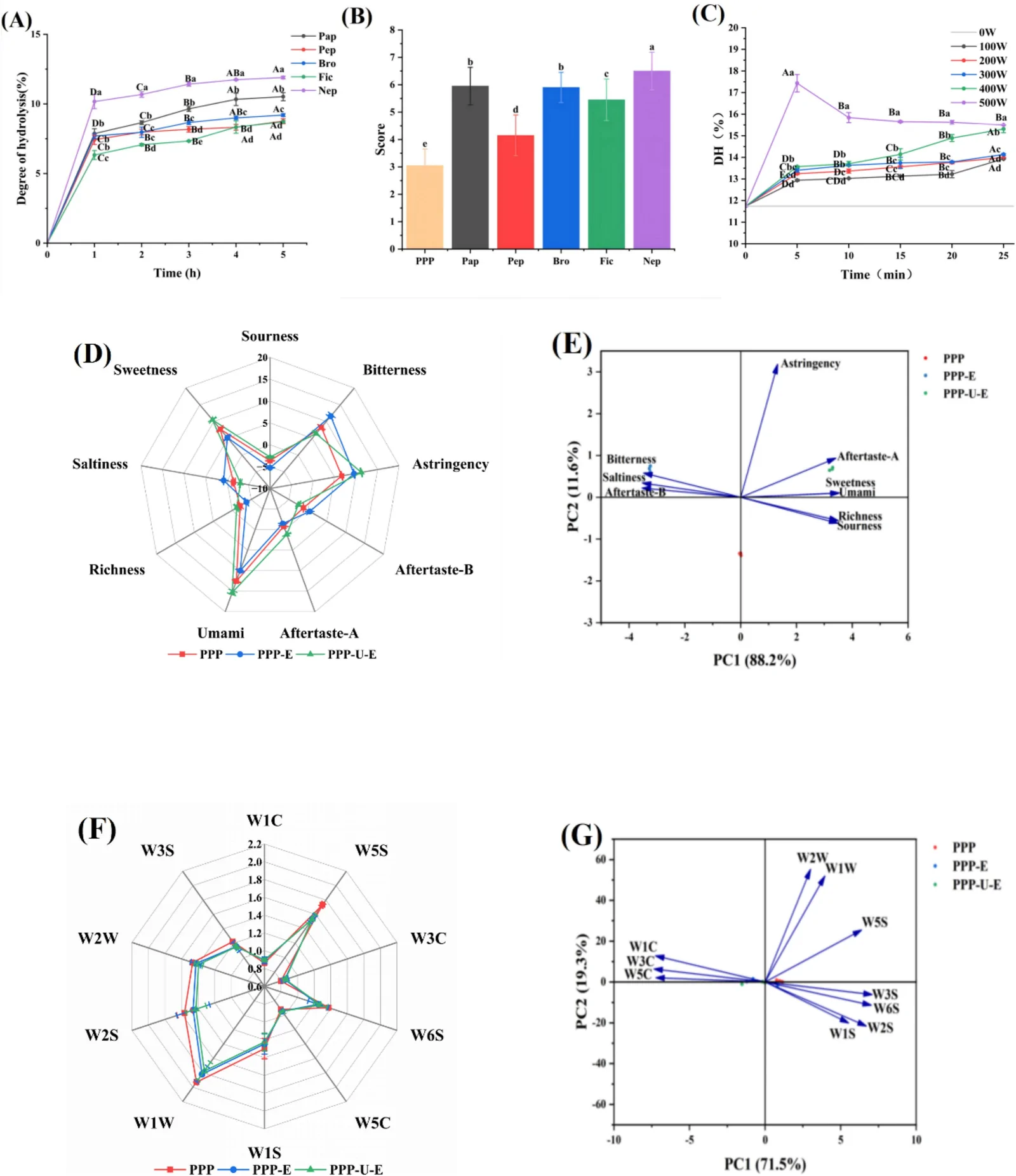
(A) Degree of hydrolysis of PPP by different proteases. (B) Sensory scores for umami. (C) Effect of ultrasonic pretreatment on the degree of hydrolysis of PPP hydrolysates. (D) Electronic tongue radar image. (E) PCA analysis of the electronic tongue. (F) Electronic nose radar image. (G) PCA analysis of the electronic nose. Significant differences (*P* < 0.05) are denoted as follows: uppercase letters for time-point comparisons within the same protease in (A) and (C); lowercase letters for inter-protease comparisons at the same time point in (A) and (C); and lowercase letters for inter-treatment comparisons in (B). Pap: papain; Pep: pepsin; Bro: stem bromelain; Fic: ficin; Nep: neutral protease; PPP: porcine plasma protein; PPP-E: enzymolyzed porcine plasma protein; PPP-U-E: ultrasound-assisted enzymolyzed porcine plasma protein. 0 W represents the control subjected to enzymatic hydrolysis only, without ultrasonic pretreatment (PPP-E). The dashed line indicates the mean DH value of the 0 W group (n = 3).

No significant increase was observed in DH after 4 h of hydrolysis (Fig. 1A). Therefore, PPP hydrolysates (PPP-E) obtained using the five proteases after 4 h were selected for sensory evaluation of umami taste. Significant differences were observed among the proteases in their ability to enhance umami intensity of PPP (*P* < 0.05; Fig. 1B). Notably, the hydrolysate prepared with Nep exhibited significantly higher umami intensity scores than those produced with the other enzymes. This result confirms the outstanding potential of Nep in enhancing the umami taste of PPP. Considering its outstanding performance in hydrolysis efficiency and umami enhancement, Nep was selected for subsequent studies.

### 3.2 Effect of ultrasonic pretreatment on the DH of PPP

Fig. 1C depicts that DH increased continuously as the ultrasonic power rose from 0 to 500 W, indicating a positive correlation between ultrasonic power and DH within this range. This can be attributed to ultrasonic pretreatment exposing additional cleavage sites in proteins, thereby facilitating more efficient enzymatic hydrolysis of peptide bonds and resulting in a higher DH [17]. A similar trend was reported by Fathi et al. [18] in their study on ultrasonic-assisted enzymatic hydrolysis of rice bran protein.

At ultrasonic powers between 0 and 400 W, DH increased significantly with prolonged treatment time (0–25 min) in all groups. However, at 500 W, DH increased sharply within the first 5 min of ultrasonic pretreatment (*P* < 0.05), reaching a maximum value of 17.43 ± 0.40% at 5 min. Beyond this point, as treatment time extended from 5 to 25 min, DH gradually declined (*P* < 0.05). These results suggest that under high ultrasonic power, a short pretreatment duration significantly enhances enzymatic hydrolysis. Excessive ultrasonic treatment may induce protein structural rearrangement or partial denaturation, reducing enzyme–substrate accessibility and thereby limiting further increases in DH [19]. The observed trend in DH is consistent with previous studies. Fadimu et al. [20] reported that lupin protein treated with ultrasonic power at 400 W exhibited an initial increase in DH followed by a slight decrease over 0 to 10 min, reaching a maximum DH (45.42%) achieved at 5 min. Similarly, Stefanović et al. [21] observed that during ultrasonic-assisted papain hydrolysis of egg white protein (34.11 ± 1.46 W), DH initially increased, followed by a decrease over 0–20 min, peaking at 39.01% at 5 min. Consequently, 5 min appears to be the optimal ultrasonic pretreatment duration for PPP hydrolysis.

Subsequently, samples pretreated with ultrasound at 500 W for 5 min (PPP-U-E) were selected for systematic comparison with PPP and PPP-E to further elucidate the mechanism by which ultrasonic-assisted enzymatic hydrolysis enhances the structural and flavor properties of PPP.

### 3.3 Electronic tongue analysis

The taste characteristics of PPP, PPP-E, and PPP-U-E were evaluated using an electronic tongue that simulates the taste mechanisms of the human tongue (Fig. 1D). Compared with PPP and PPP-E, PPP-U-E exhibited significantly higher responses for umami and sweetness (*P* < 0.05). These results indicate that ultrasonic pretreatment can enhance flavor compound formation, which is considered desirable. This enhancement is most likely due to ultrasound-induced protein denaturation, which helps release umami-rich amino acids such as glutamic acid and aspartic acid, as well as sweet-tasting amino acids such as glycine and alanine [22]. However, the bitterness response value of PPP-E increased relative to that of PPP, which can be attributable to exposure of hydrophobic peptide segments generated during enzymatic hydrolysis. Du et al. [23] reported a similar phenomenon during the enzymatic hydrolysis of shiitake mushrooms.

To comprehensively evaluate differences in taste profiles, electronic tongue data were further analyzed using PCA. The three sample groups formed distinct, non-overlapping clusters in the PCA score plot (Fig. 1E). PC1 and PC2 accounted for 99.8% of the total variance (> 85%), suggesting they effectively represent inter-sample flavor variations. Compared with PPP and PPP-E groups, the PPP-U-E group was clearly positioned along the positive axis of PC1, which correlated with the umami and sweetness directions. These results further support the hypothesis that ultrasonic pretreatment before enzymatic hydrolysis enhances the umami and sweet taste attributes of PPP.

### 3.4 Electronic nose analysis

Flavor characteristics of the sample groups were evaluated using an electronic nose. PPP-E and PPP-U-E exhibited significantly lower response values for the W5S sensor (nitrogen oxides) than PPP (*P* < 0.05; Fig. 1F). This indicates that enzymatic hydrolysis and ultrasonic-assisted treatment effectively reduce undesirable odors associated with nitrogen-containing compounds. Weng et al. [24] reported that moderate hydrolysis of soybean meal enhances flavor stability, whereas excessive hydrolysis may lead to nitrogenous off-flavor substance accumulation.

Furthermore, the overall flavor radar profile of PPP-U-E contracted toward the center compared with PPP and PPP-E groups, suggesting that ultrasonic-assisted enzymatic hydrolysis reduces the response of other off-flavor compounds, yielding a more balanced and milder overall flavor profile. Notably, Sun et al. [25] observed that flavor deterioration in stored sea cucumber was accompanied by an expansion of the electronic nose radar profile, with increased responses for nitrogen-containing (W5S) and sulfur-containing (W1W) compounds. This observed expansion contrasts with the contraction observed in this study, indirectly supporting the positive effect of ultrasonic-assisted enzymatic hydrolysis in improving the flavor of PPP.

PCA of the electronic nose response values showed that PC1 and PC2 explained 71.5% and 19.3% of the variance, respectively, with a cumulative contribution of 90.8% (Fig. 1G). These results indicate that PCA effectively captures the overall flavor information and exhibits high reliability. The vector corresponding to the W5S sensor exhibited a strong positive correlation with the PC1 axis and pointed toward the region corresponding to the PPP sample. This finding indicates that the off-flavor of the PPP sample is primarily associated with nitrogen oxides. Conversely, PPP-E and PPP-U-E treatment groups shifted in the direction opposite to the W5S vector, demonstrating that enzymatic hydrolysis and ultrasonic-assisted enzymatic hydrolysis effectively reduce key off-flavor compounds.

### 3.5 Structural changes of PPP after ultrasonic pretreatment and enzymolysis

#### 3.5.1 Particle size distribution and zeta potential

To elucidate the physicochemical basis by which ultrasound-assisted enzymolysis improves PPP flavor, the average particle size and zeta potential of the sample were measured (Fig. 2A). The native PPP exhibited a relatively large average particle size of 298.07 ± 5.12 nm and a zeta potential of –11.43 ± 1.16 mV, indicating the presence of large aggregates and weak electrostatic repulsion between particles. Enzymatic hydrolysis significantly reduced the particle size of PPP-E to 215.67 ± 3.68 nm, whereas the absolute value of the zeta potential (–17.76 ± 0.74 mV) significantly increased (*P* < 0.05). These changes demonstrate that enzymatic hydrolysis effectively disrupted PPP aggregates and exposed additional ionizable groups. When ultrasonic-assisted enzymatic hydrolysis was applied, the particle size of PPP-U-E further decreased significantly to 160.37 ± 2.06 nm, whereas the zeta potential remained at a high absolute value (–17.98 ± 0.50 mV). These changes indicate that ultrasonication enhances particle stability and diffusivity of unfolded protein or peptide structures in the PPP hydrolysis products. This finding is consistent with the observations of Yang et al. [26]. The combined decrease in particle size and increase in the absolute value of the zeta potential suggest that enzymatic hydrolysis and ultrasonic pretreatment play a crucial regulatory role in flavor modification of PPP. The observed decrease in particle size is likely attributable to the combined effect of ultrasonic cavitation and enzymatic hydrolysis. This interplay enhances the accessibility of protein structures, which in turn facilitates the release of flavor-active peptides and amino acids [27]. Furthermore, the increased absolute value of the zeta potential reflects enhanced system stability, which may enable stable dispersion of flavor-active compounds within the system [28].

**Fig. 2:**
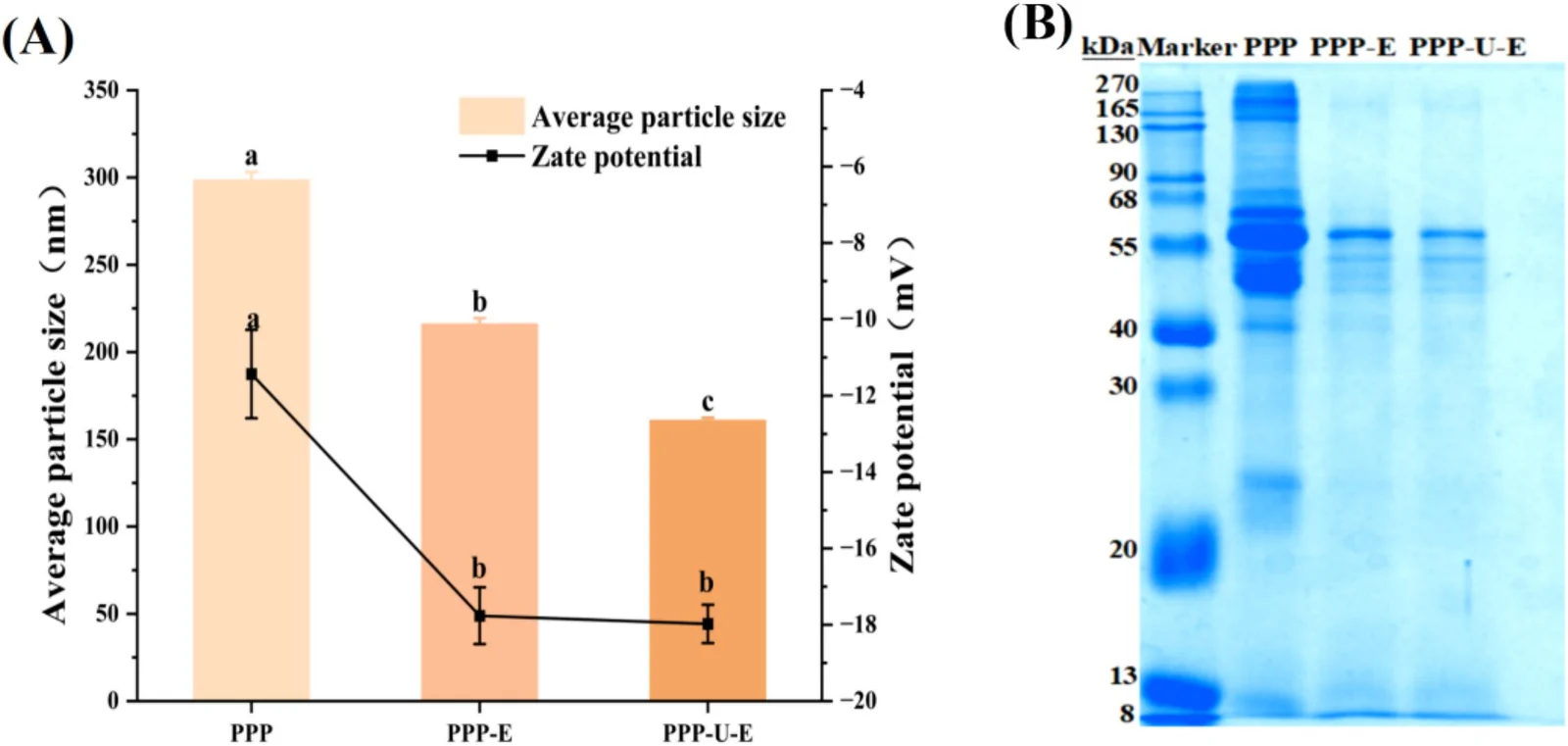
(A) Particle size and zeta potential. (B) SDS-PAGE. Different letters in (A) indicate significant differences among treatment groups (*P* < 0.05). PPP: porcine plasma protein; PPP-E: enzymolyzed porcine plasma protein; PPP-U-E: ultrasound-assisted enzymolyzed porcine plasma protein.

#### 3.5.2 SDS-PAGE

The molecular weight distribution was subsequently assessed using SDS-PAGE (Fig. 2B). PPP exhibited a typical plasma protein profile, including distinct bands for albumin (66 kDa), β-globulin (48 kDa), and α-globulin (56 kDa), consistent with the characteristic bands reported by Samsalee et al. [2], confirming the structural integrity of the untreated protein. Conversely, bands above 55 kDa were nearly absent in PPP-E and PPP-U-E, whereas pronounced diffuse bands appeared in the low-molecular-weight region (<13 kDa). These findings signify that PPP was extensively hydrolyzed into small peptides under the combined action of enzymatic digestion and ultrasonication. This pattern aligns with the diffuse bands observed below 20 kDa by Kumari et al. [29] in their study on sheep plasma protein hydrolysis. Notably, compared with PPP-E, the electrophoretic bands of PPP-U-E shifted further toward the <10 kDa region, indicating that ultrasonic pretreatment promoted more extensive protein hydrolysis. Ultrasonication-induced cavitation likely drives this enhancement through improved enzyme–substrate interactions [30]. These findings are consistent with the higher degree of hydrolysis observed in the PPP-U-E group (Fig. 1C).

#### 3.5.3 Secondary structure

FTIR spectroscopy was used to characterise the vibrational features of functional groups and changes in protein secondary structure. No novel characteristic absorption peaks were identified in the FTIR spectra of PPP-E and PPP-U-E compared to those of PPP (Fig. 3A).

**Fig. 3:**
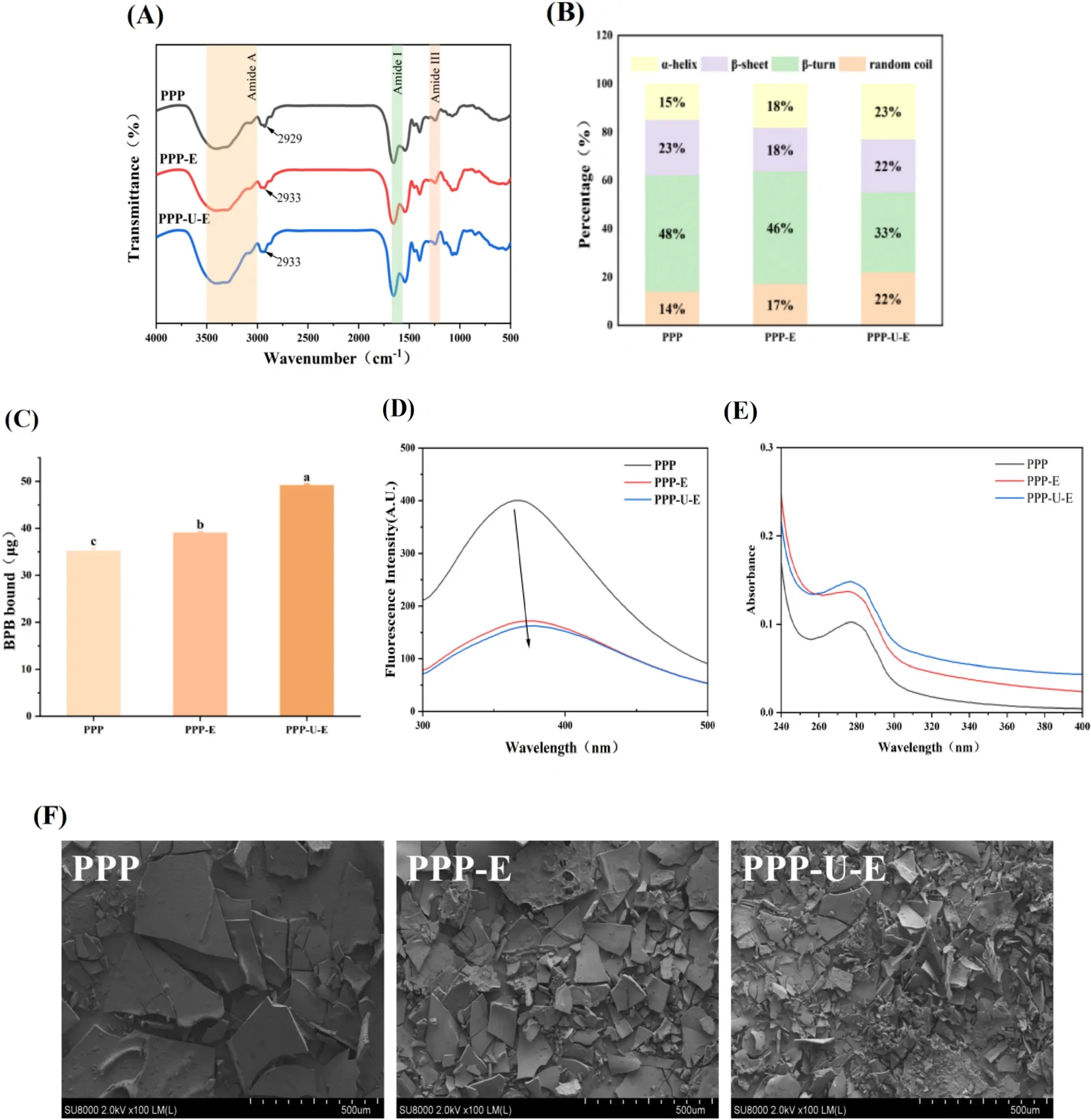
(A) FTIR spectra. (B) Secondary structure changes. (C) Surface hydrophobicity. (D) Intrinsic fluorescence spectroscopy. (E) UV–Vis absorption spectra. (F) Scanning electron microscopy. Different lowercase letters in (C) indicate significant differences among treatments (*P* < 0.05). PPP: Porcine plasma protein; PPP-E: Enzymatically hydrolyzed porcine plasma protein; PPP-U-E: Ultrasound-assisted enzymatically hydrolyzed porcine plasma protein.

The absorption peaks in the amide A band (3500–3000 cm^−1^) primarily arise from N–H and O–H stretching vibrations in the peptide backbone. Variations in peak shape and position directly reflect the stability of intramolecular and intermolecular hydrogen bonds [14]. The amide I band (1700–1600 cm^−1^) arises primarily from C=O stretching vibrations and is a key marker of protein secondary structure [2]. The amide III band (1320–1200 cm^−1^) primarily corresponds to C–N stretching and N–H bending vibrations [31]. The hydrophobic region structure of proteins can be inferred from C–H stretching vibrations of aliphatic side chains (3000–2800 cm^−1^) [32]. Compared with the C–H stretching peak of PPP (2929 cm^−1^), PPP-E and PPP-U-E exhibited a blue shift, indicating structural alterations in the hydrophobic regions of PPP. It has been reported that the vibrational modes of functional groups significantly influence protein secondary structure formation [33]. Consequently, the observed blue shift in the C–H vibrational peaks further corroborates conformational rearrangement within the secondary structure of PPP.

A quantitative analysis of the amide I band (1600–1700 cm^−1^) was conducted using PeakFit software to determine the secondary structure composition of the three sample groups. Compared with PPP, the α-helix and random coil contents in PPP-E and PPP-U-E groups exhibited a significant increase, whereas the β-sheet content demonstrated a significant decrease (Fig. 3B). These results indicate that enzymatic hydrolysis and ultrasonication promote a transformation of the protein secondary structure from β-sheets to α-helices and random coils. Among the groups, the PPP-U-E group exhibited the highest α-helix and random coil contents (23% and 22%, respectively) and the lowest β-turn content (33%), indicating that ultrasonic-assisted enzymatic hydrolysis exerts a more pronounced modulatory effect on protein secondary structure. Combined with the absence of new characteristic absorption peaks in the FTIR spectra (Fig. 3A), these results suggest that enzymatic hydrolysis and ultrasonication mainly induced physical structural rearrangements in PPP.

The observed conformational changes can be attributed to ultrasonic cavitation, which disrupts hydrogen bonds and hydrophobic interactions within protein molecules. This effect enhances the unfolding of ordered structures, resulting in a more loosely organized, overall disordered protein conformation [34]. Consistent with these findings, Hu et al. [35] reported that soybean protein isolate subjected to ultrasonic treatment (400 W for 30 min and 600 W for 30 min) exhibited increased α-helix and random coil contents and decreased β-sheet content compared with the control group. Zhou et al. [36] reported that increased α-helix content in proteins exposes internal hydrophobic sites, enhancing surface hydrophobicity. Likewise, Tian et al. [37] observed that α-helix enrichment during ultrasound-assisted hydrolysis of coconut protein improved protein rigidity and stability.

#### 3.5.4 Tertiary structure

A subsequent analysis of surface hydrophobicity (Fig. 3C) revealed that PPP-U-E and PPP-E groups exhibited significantly higher surface hydrophobicity than that of the PPP group (*P* < 0.05). These findings indicate that enzymatic hydrolysis and ultrasonic pretreatment effectively disrupt the tightly folded protein conformation, exposing previously buried hydrophobic groups. Additionally, the surface hydrophobicity of the PPP-U-E group was significantly higher than that of the PPP-E group, thereby substantiating the hypothesis that ultrasonic pretreatment enhances PPP enzymatic hydrolysis. These findings are consistent with those of Dong et al. [38], who reported that ultrasound-assisted treatment of chicken bone protein markedly increased surface hydrophobicity of protein hydrolysates compared with enzymatic hydrolysis alone.

Intrinsic fluorescence spectroscopy, which detects fluorescence signals from aromatic amino acids (phenylalanine, tryptophan, and tyrosine), is highly sensitive to changes in protein tertiary structure [39]. The fluorescence intensities of PPP-E and PPP-U-E were significantly diminished across the 300–500 nm range compared to those of PPP (Fig. 3D). The findings suggest that enzymatic hydrolysis and ultrasonication effectively induce peptide chain unfolding and expose originally buried fluorescence-sensitive groups within the molecule to the solvent. The decrease in fluorescence intensity likely results from changes in the distance or orientation between tryptophan and tyrosine residues, leading to reduced energy transfer efficiency [30]. Concurrently, protein conformational unfolding increases the accessibility of fluorescence-quenching groups, thereby further enhancing the fluorescence-quenching effect [40]. Notably, the maximum wavelength values for PPP, PPP-E, and PPP-U-E were 366, 373, and 378 nm, respectively. A comparison of PPP, PPP-E, and PPP-U-E revealed red shifts of 7 and 12 nm, respectively. These results further suggest that sonication-assisted proteolysis facilitates aromatic amino acid exposure within the protein. This phenomenon was observed in a study by Liu et al. [31] on black sesame protein hydrolysates, where all proteolyzed samples exhibited fluorescence quenching accompanied by red shifts.

UV–Vis absorption spectra (Fig. 3E) further support the observed conformational changes. The characteristic protein absorption peak at 280 nm arises primarily from aromatic amino acid residues [41]. The intensity of this characteristic absorption peak was significantly higher in PPP-E and PPP-U-E groups compared with the PPP group. The PPP-U-E group exhibited the highest intensity at 280 nm. These results further confirm that ultrasound-assisted enzymolysis promotes PPP’s conformational unfolding. Meanwhile, the unchanged absorption peak position suggests a dynamic rather than static quenching mechanism [42].

Overall, analysis of the tertiary structure indicates that ultrasonic-assisted enzymatic hydrolysis significantly enhances PPP unfolding, leading to substantial exposure of hydrophobic regions and aromatic groups. These structural changes likely underpin the enhanced umami taste observed in the electronic tongue analysis.

#### 3.5.5 SEM

SEM revealed microstructural changes in PPP. Fig. 3F depicts that PPP exhibited a dense, continuous lamellar structure, indicative of its native, intact conformation. However, PPP-E exhibited a fragmented, porous morphology, suggesting that enzymatic hydrolysis disrupted the protein’s backbone. Notably, PPP-U-E exhibited a more extensive, fragmented, loose, and porous architecture, reflecting greater extensive structural disruption. This progressive increase in structural loosening and porosity can be attributed to the combined effects of enzymatic hydrolysis and ultrasonic cavitation, including microjets and turbulent forces generated during ultrasonic treatment [43]. A similar observation was reported by Yang et al. [26] in their study on housefly larval proteins, where ultrasonic-assisted enzymatic hydrolysis produced more uneven, finer particles than enzymatic hydrolysis alone.

### 3.6 Free amino acid (FAA) content

FAAs are key contributors to characteristic food flavors [44]. Here, 17 FAAs were quantified, including umami amino acids (Glu, Asp), sweet amino acids (Thr, Ser, Gly, Ala, Arg, and Pro), and bitter amino acids (Val, Met, Ile, Leu, Phe, Tyr, Lys, and His). PPP-E and PPP-U-E exhibited significantly higher total FAA content compared with PPP (*P* < 0.05; Fig. 4A). The increase in FAAs in PPP-E can be primarily attributed to the cleavage of peptide bonds during enzymatic hydrolysis [7]. Notably, the PPP-U-E group exhibited the highest total FAA content (3515.81 ± 12.17 μg/g), representing increases of 146.99% and 24.93% compared with the PPP and PPP-E groups, respectively. This finding indicates a combined effect of ultrasonication and enzymatic hydrolysis in promoting amino acid release. This trend is consistent with the findings of Zhang et al. [45], who reported that ultrasonic pretreatment followed by enzymatic hydrolysis exhibited the highest total FAA content (1278.3 mg/100 g).

**Fig. 4:**
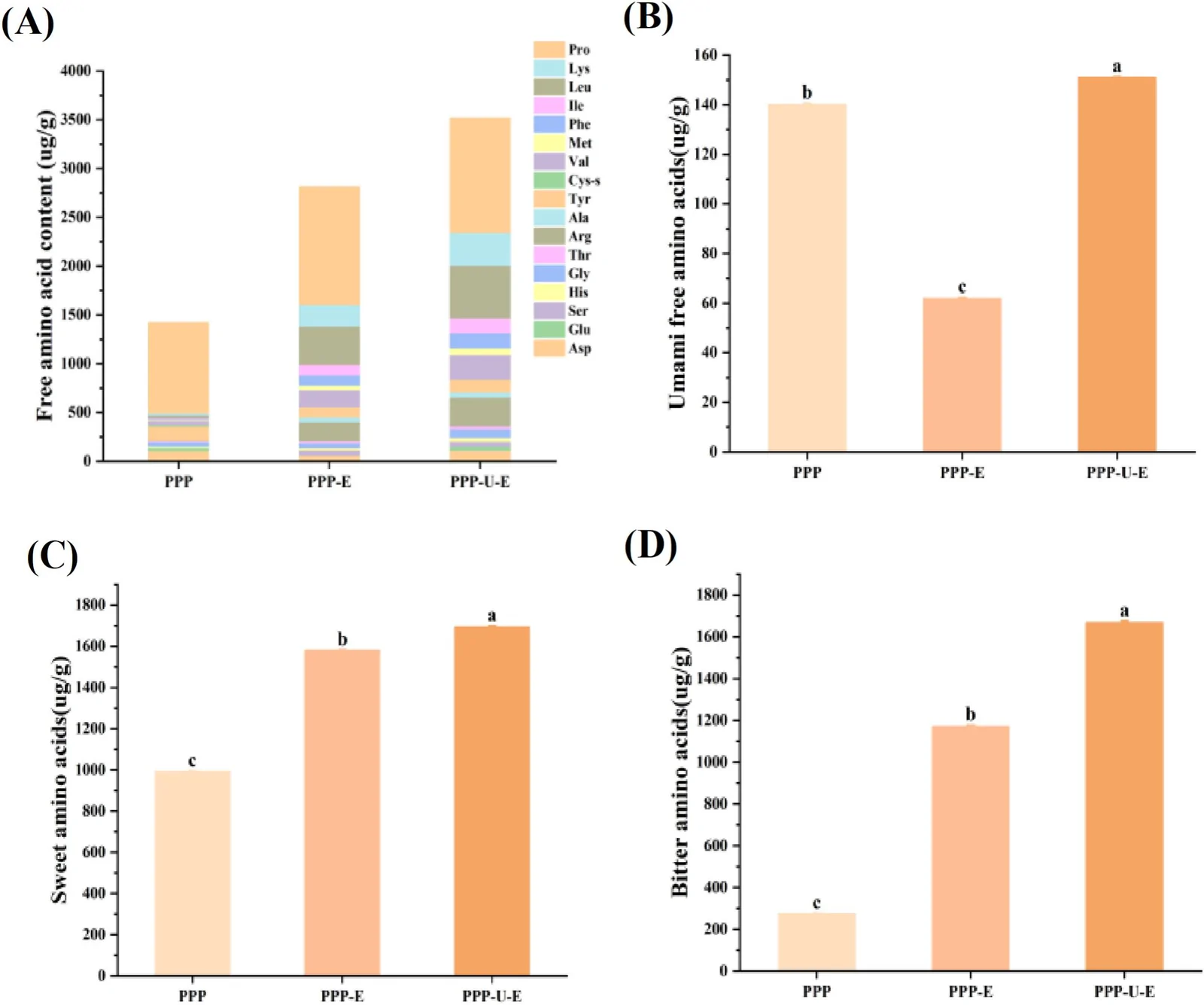
(A) Free amino acid content. (B) Umami amino acid content. (C) Sweet amino acid content. (D) Bitter amino acid content. Different letters indicate significant differences among treatment groups (*P* < 0.05). PPP: Porcine plasma protein; PPP-E: Enzymatically hydrolyzed porcine plasma protein; PPP-U-E: Ultrasound-assisted enzymatically hydrolyzed porcine plasma protein.

Umami and sweet amino acids levels in the PPP-U-E group were significantly higher than those in the PPP group (*P* < 0.05; Fig. 4B and C). This suggests that ultrasonic-assisted enzymatic hydrolysis selectively enhances the release of umami and sweet compounds, thereby contributing to the harmonization and overall flavor enhancement. However, bitter amino acids increased in PPP-E and PPP-U-E groups (Fig. 4D), indicating that although sonication-assisted enzymatic hydrolysis optimizes flavor, it simultaneously releases specific hydrophobic peptide fragments and bitter amino acids. Similarly, Zhang et al. [45] reported that in oyster hydrolysates, ultrasonic-assisted enzymolysis promoted FAA release but also increased the levels of hydrophobic amino acids, contributing to bitterness development.

### 3.7 Sensory evaluation

Fig. 5 depicts the sensory evaluation results. PPP-U-E exhibited significantly higher umami and sweetness scores than PPP and PPP-E, along with a significantly lower bitterness score, and achieved the highest overall acceptability (*P* < 0.05). These findings indicate that PPP-U-E possesses a well-balanced flavor profile, characterized by dominant umami and sweetness notes, with relatively weak bitterness perception.

**Fig. 5:**
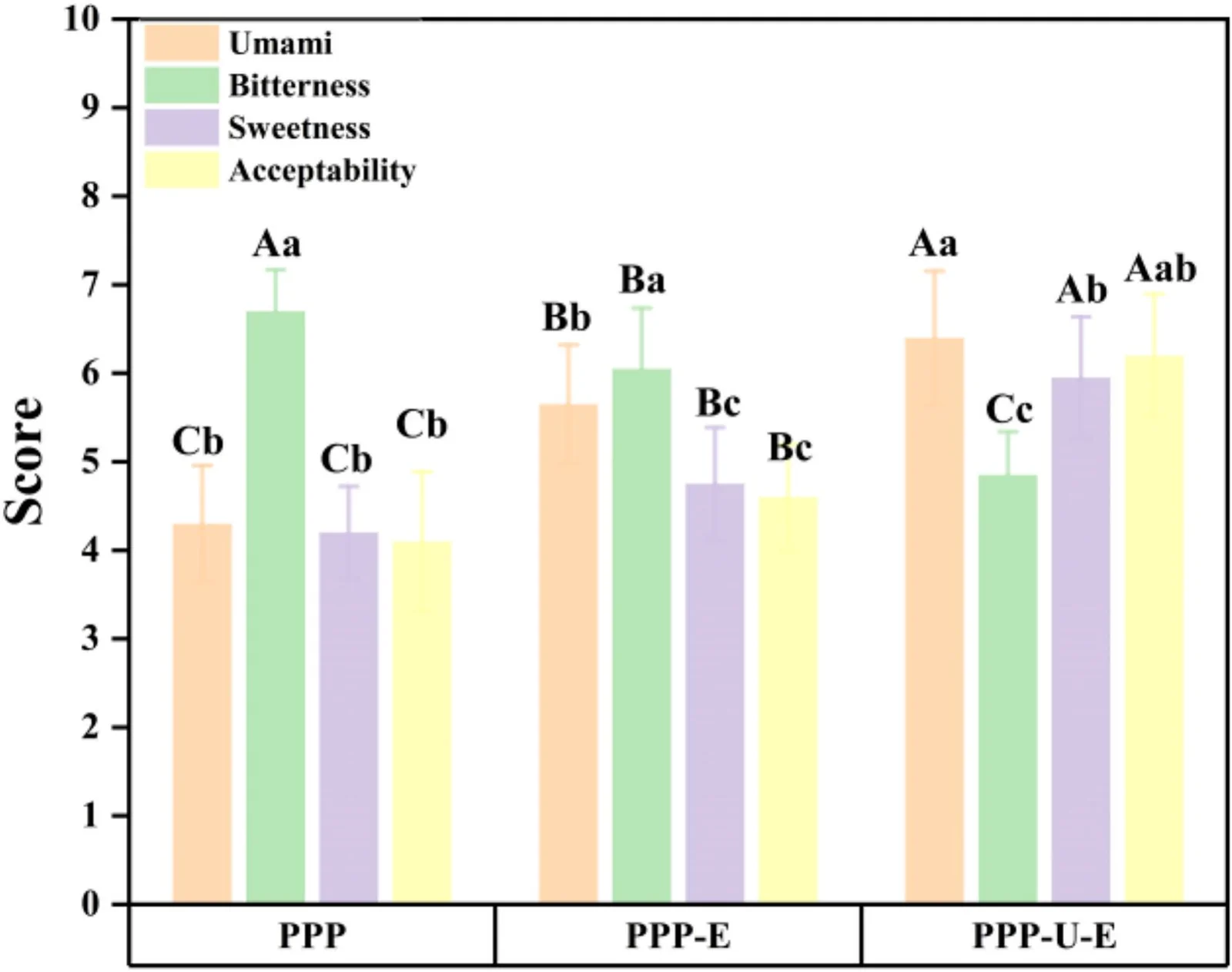
Sensory evaluation. Different uppercase letters indicate significant differences between treatment groups; different lowercase letters indicate significant differences among sensory parameters within a group (*P* < 0.05). PPP: porcine plasma protein; PPP-E: enzymatically hydrolyzed porcine plasma protein; PPP-U-E: ultrasonic-assisted enzymatically hydrolyzed porcine plasma protein.

The sensory findings are consistent with instrumental sensory assessment data. Electronic tongue analysis (Fig. 1D) revealed significantly increased umami and sweetness response in PPP-U-E. Free amino acid analysis further confirmed that PPP-U-E contained the highest levels of umami and sweet amino acids, providing a compositional basis for attributes observed in the sensory evaluation. Although bitter amino acids also increased in PPP-U-E (Fig. 4D), no corresponding increase in perceived intensity of bitterness was observed during the sensory evaluation. This discrepancy may be attributed to flavor interactions among amino acids that partially mask or offset bitterness perception [46]. Electronic nose analysis (Fig. 1F) indicated that ultrasonic-assisted enzymatic hydrolysis attenuates undesirable odor components, consistent with the lower bitterness scores and higher overall acceptability observed in the sensory evaluation.

Collectively, these results demonstrated that ultrasonic-assisted enzymatic hydrolysis has a combined effect in enhancing the umami and sweetness of porcine plasma protein hydrolysates and suppressing bitterness and undesirable odors, thereby significantly improving overall flavor quality and consumer acceptability.

## 4 Conclusions

This study demonstrated that ultrasound-assisted enzymatic hydrolysis can effectively modulate PPP conformation. This process significantly enhances hydrolysis efficiency and improves flavor characteristics. Ultrasound pretreatment (500 W, 5 min) combined with neutral protease hydrolysis is an effective method for preparing protein hydrolysate with a high degree of hydrolysis (17.43 ± 0.40%) and a favorable flavor profile. Structural analyses revealed that ultrasound-assisted enzymatic hydrolysis promoted extensive denaturation of the protein’s tertiary structure and rearrangement of its secondary structure, specifically manifested by a significant increase in the α-helix and random coil contents (23% and 22%, respectively) and a significant decrease in the β-turn content (33%). Free amino acid analysis further demonstrated that ultrasonic-assisted enzymatic hydrolysis significantly facilitated the release of flavor-contributing amino acids, with umami amino acid content reaching 151.06 ± 0.21 μg/g and sweet amino acid content reaching 1696.04 ± 4.54 μg/g. These findings provide the foundation for imparting an ideal savory-sweet flavor to the product.

These findings were corroborated by electronic nose, electronic tongue, and sensory evaluations, which further confirmed that ultrasonic-assisted enzymatic hydrolysis significantly improved the overall flavor and sensory acceptability of the product. In summary, this technology represents an effective approach for enhancing the flavor of pig plasma protein and promoting its high-value applications, providing theoretical and technical foundation for developing flavor-enhanced protein ingredients. In addition, further identification of the specific taste-active peptides generated during ultrasound-assisted enzymatic hydrolysis would help clarify the molecular basis of flavor enhancement. Future studies could also evaluate the application potential of the resulting hydrolysates in real food systems.

## CRediT authorship contribution statement

**Lingrui Li:** Writing – original draft. **Jingwei Pan:** Investigation, Data curation. **Tiantian Wu:** Investigation, Data curation. **Chuan Pang:** Investigation, Data curation. **Xin Guo:** Investigation, Data curation. **Shiling Lu:** Writing – review & editing. **Jingyun Wang:** Writing – review & editing.

## Declaration of competing interest

The authors declare that they have no known competing financial interests or personal relationships that could have appeared to influence the work reported in this paper.

## Appendix A Supplementary data

Supplementary Data 1 E-nose and E-tongue data.
