# Supplementary material for: Modification of porcine plasma protein structure and flavor by ultrasound-assisted enzymatic hydrolysis
Source: Ultrason Sonochem. 2026 Oct 12;134:108111. doi: 10.1016/j.ultsonch.2026.108111 (PMC13691553; doi:10.1016/j.ultsonch.2026.108111)
Supplement: Supplementary file 1 [file mmc1.docx]

**Supplementary Material**

**Table S1.** Electronic tongue sensor response values of different treatment groups.

| Sensor response value | PPP | PPP-E | PPP-U-E |
| --- | --- | --- | --- |
| Sweetness | 7.70±0.18^b^ | 5.20±0.13^c^ | 10.50±0.04^a^ |
| Sourness | -3.64±0.06^b^ | -5.24±0.06^c^ | -2.81±0.04^a^ |
| Bitterness | 8.25±0.06^b^ | 11.61±0.38^a^ | 6.40±0.12^c^ |
| Astringency | 6.67±0.08^c^ | 9.51±0.09^b^ | 11.21±0.07^a^ |
| Aftertaste-B | -1.18±0.01^b^ | 0.50±0.01^a^ | -2.53±0.06^c^ |
| Aftertaste-A | -0.80±0.01^c^ | -1.47±0.03^b^ | 1.20±0.04^a^ |
| Umami | 12.52±0.05^b^ | 9.99±0.11^c^ | 15.31±0.43^a^ |
| Richness | -2.10±0.05^b^ | -3.78±0.08^c^ | -1.16±0.03^a^ |
| Saltiness | -1.50±0.03^b^ | 0.80±0.01^a^ | -3.13±0.04^c^ |

Data are presented as mean ± SD (n = 3). Different superscript lowercase letters within the same row indicate statistically significant differences among groups (*P* < 0.05).

**Table S2.** Electronic nose sensor response values of different treatment groups.

| Sensor response value | PPP | PPP-E | PPP-U-E |
| --- | --- | --- | --- |
| W1C | 0.86±0.01^a^ | 0.89±0.04^a^ | 0.90±0.02^a^ |
| W5S | 1.73±0.03^a^ | 1.54±0.05^b^ | 1.53±0.07^b^ |
| W3C | 0.80±0.01^a^ | 0.85±0.05^a^ | 0.85±0.03^a^ |
| W6S | 1.37±0.02^a^ | 1.25±0.12^a^ | 1.28±0.08^a^ |
| W5C | 0.92±0.00^a^ | 0.94±0.02^a^ | 0.95±0.01^a^ |
| W1S | 1.30±0.11^a^ | 1.25±0.11^a^ | 1.22±0.10^a^ |
| W1W | 1.93±0.02^a^ | 1.82±0.11^a^ | 1.78±0.12^a^ |
| W2S | 1.57±0.09^a^ | 1.46±0.20^a^ | 1.43±0.12^a^ |
| W2W | 1.47±0.00^a^ | 1.43±0.06^a^ | 1.40±0.05^a^ |
| W3S | 1.22±0.01^a^ | 1.15±0.06^a^ | 1.15±0.03^a^ |

Data are presented as mean ± SD (n = 3). Different superscript lowercase letters within the same row indicate statistically significant differences among groups (*P* < 0.05).
